# The Cardiac Dysfunction Caused by Metabolic Alterations in Alzheimer's Disease

**DOI:** 10.3389/fcvm.2022.850538

**Published:** 2022-02-22

**Authors:** Jiayuan Murphy, Tran Ngoc Van Le, Julia Fedorova, Yi Yang, Meredith Krause-Hauch, Kayla Davitt, Linda Ines Zoungrana, Mohammad Kasim Fatmi, Edward J. Lesnefsky, Ji Li, Di Ren

**Affiliations:** ^1^Department of Surgery, Morsani College of Medicine, University of South Florida, Tampa, FL, United States; ^2^Pauley Heart Center, Division of Cardiology, Department of Internal Medicine, Virginia Commonwealth University, Richmond, VA, United States; ^3^Cardiology Section, Medical Service, Richmond Department of Veterans Affairs Medical Center, Richmond, VA, United States

**Keywords:** Alzheimer's Disease, cardiac dysfunction, mitochondrial deficits, metabolic regulation, glucose metabolic alterations

## Abstract

A progressive defect in the energy generation pathway is implicated in multiple aging-related diseases, including cardiovascular conditions and Alzheimer's Disease (AD). However, evidence of the pathogenesis of cardiac dysfunction in AD and the associations between the two organ diseases need further elucidation. This study aims to characterize cellular defects resulting in decreased cardiac function in AD-model. 5XFAD mice, a strain expressing five mutations in human APP and PS1 that shows robust Aβ production with visible plaques at 2 months and were used in this study as a model of AD. 5XFAD mice and wild-type (WT) counterparts were subjected to echocardiography at 2-, 4-, and 6-month, and 5XFAD had a significant reduction in cardiac fractional shortening and ejection fraction compared to WT. Additionally, 5XFAD mice had decreased observed electrical signals demonstrated as decreased R, P, T wave amplitudes. In isolated cardiomyocytes, 5XFAD mice showed decreased fraction shortening, rate of shortening, as well as the degree of transient calcium influx. To reveal the mechanism by which AD leads to cardiac systolic dysfunction, the immunoblotting analysis showed increased activation of AMP-activated protein kinase (AMPK) in 5XFAD left ventricular and brain tissue, indicating altered energy metabolism. Mito Stress Assays examining mitochondrial function revealed decreased basal and maximal oxygen consumption rate, as well as defective pyruvate dehydrogenase activity in the 5XFAD heart and brain. Cellular inflammation was provoked in the 5XFAD heart and brain marked by the increase of reactive oxygen species accumulation and upregulation of inflammatory mediator activities. Finally, AD pathological phenotype with increased deposition of Aβ and defective cognitive function was observed in 6-month 5XFAD mice. In addition, elevated fibrosis was observed in the 6-month 5XFAD heart. The results implicated that AD led to defective mitochondrial function, and increased inflammation which caused the decrease in contractility of the heart.

## Introduction

Alzheimer's Disease (AD) is a common aging-related neurological disorder that manifests as impaired cognition, short-term memory loss, increased irritability, and personality changes (1, 2). The clinical conditions of AD are contributed to neuronal death that leads to an overall reduction of brain volume, decreased metabolic capacity and glucose utilization as well as decreased synaptic transmissions (3, 4). Although the molecular hypothesis of disease generation is still under intensive investigation, the two most prominent hypotheses suggest these associated neuronal damages are caused by intracellular tau aggregation and extracellular beta-amyloid (Aβ) deposition, which interrupts normal metabolic processes and leads to neuronal apoptosis (5).

AD has numerous comorbidities, including several major cardiovascular diseases (4, 6, 7). The interplay amongst these conditions is not yet clearly defined. However, they share one glaring similarity characterized by a significant increase in disease incidences onset in the elderly population, and aging is associated with worsening disease progression (8, 9). With aging, one notable change is the reduction of energy uptake and utilization (10). This metabolic impairment is both heavily implicated in AD and cardiovascular dysfunction (11, 12). It has been proposed that the systematic reduction of metabolic activities and the consequences following are prominent causes for many age-related diseases, including AD and cardiovascular disorders (13).

Although aging is the single most important risk factor in AD, the disease itself is characterized by a pathological acceleration of the aging process of the brain, which is the development of increased beta-amyloid load, neuronal degeneration, reduction of brain volume, and cognitive impairment (14). One of the hallmarks of AD, increased extracellular deposition of Aβ and thus the increase in Aβ in systematic circulation can also affect the heart, another highly perfused organ, by interrupting the metabolic processes of cardiomyocytes and therefore leads to cardiovascular diseases (5). This process links the acceleration in aging of the central nervous system with the cardiovascular system; in turn, the worsened cardiac function and metabolic processes create a vicious cycle that feeds back to the cerebral region with a decreased concentration in the substrate neurons require for energy generation, which further worsens the symptoms seen in AD (5).

Amongst the concurrent conditions AD is associated with, cardiovascular injury has been observed as experimental evidence. For example, Tg2576 mice which express a highly elevated level of APP is associated with the development of cognitive impairment and ROS-mediated endothelial dysfunction with perfusion reduction (2, 15). APP23 mice, which also overexpress APP and Ab1-40, are prone to dementia and when crossed with apolipoprotein E–deficient (ApoE^−/−^) mice, develop exacerbated aortic atherosclerotic lesions compared to ApoE^−/−^ (16). The evidence suggests a clear link between the etiology of AD and cardiovascular dysfunction. In this study, we aim to address the effect intrinsic to cardiac function associated with AD and systematically classify the detailed impairment.

To achieve the goal of further illustrating the pathogenesis of cardiovascular dysfunction related to AD, we demonstrate in this study that a strain of AD model mice, 5XFAD mice, has significantly worse cardiac outcomes such as decreased contractility and increased inflammatory markers caused by damage to mitochondrial function, possibly related to increased Aβ plaque deposition. 5XFAD mice overexpress the K670N/M671L (Swedish), I716V (Florida), and V717I (London) mutations in human APP (695), as well as M146L and L286V mutations in human PS1. Aβ plaques deposition is observed in these mice as early as at 2-months of age and is associated with progressively worsening cognitive status seen in behavioral studies, which are consistent findings in AD in the human. Therefore, 5XFAD mice are highly useful tools for studying AD-related diseases.

## Materials and Methods

### Animals

5XFAD and wild type (WT) (2–6 months) C57BL/6J mice were bred and supplied by our lab as the previous reports described (17–19). All animal protocols in this study were approved by the Institutional Animal Care and Use Committee of the University of South Florida and conform to the NIH Guide for the care and use of laboratory animals.

### *In vivo* Cardiac Function Evaluation by Echocardiography

5XFAD and WT mice of 2-, 4-, and 6-months old were subjected to trans-thoracic M-mode and Doppler mode echocardiography using the FUJIFILM VisualSonic Vevo 3100 system. The cardiac systolic and diastolic functions were assessed using the previously described protocol (20). Simpson's measurements were performed to obtain the systolic function features, such as calculated averaged ejection fraction (EF) and fractional shortening (FS) (20, 21).

### *In vivo* Cardiac Function Assessment by Electrocardiography

At age of 2-, 4-, and 6-months, changes in the electrical activity of the myocardium in 5XFAD and WT mice were detected by the electrocardiogram (ECG) in Lead II (Mac Lab/4E ECG module, AD Instruments). P amplitude, R amplitude, T amplitude, PR interval, QT interval, and Tpeak-Tend were obtained and analyzed with software LabChart of mouse model default setting from AD instrument.

### Cardiomyocyte's Isolation

Heparin IV (Fresenius Kabi) for anticoagulation was given by intraperitoneal injection with 1,000 units/kg 10 min before the experiment (22). Six-month old 5XFAD and WT mice underwent anesthesia with 2–3% isoflurane and 100% O_2_. The hearts of mice were excised, then cannulated by the aorta, and connected to the cardiomyocyte perfusion apparatus (Radnoti). The heart was perfused at 37°C with a Ca^2+^ free based buffer (pH 7.2) containing: 135 mM NaCl, 4 mM KCl, 1 mM MgCl_2_, 10 mM HEPES, 0.33 mM NaH_2_PO_4_, 10 mM glucose, 10 mM 2, 3-butanedione monoxime, and 5 mM taurine that was bubbled with O_2_. The heart was digested with 0.03 mg/ml Liberase (Sigma, # 5401020001) dissolved in perfusion buffer. After digested completely, the heart was removed, torn with tweezers, and blown gently, then filtered to obtain the isolated cardiomyocytes.

### Measurement of Contractility of the Cardiomyocytes

The contractile properties of cardiomyocytes were assessed by an IonOptix Multi Cell High Throughput system (IonOptix Corporation). Cardiomyocytes were placed in a chamber and stimulated with a 14-voltage at a frequency of 1 Hz. IonWizard software was used to record the changes in sarcomere length and duration of shortening and relengthening. The following parameters were used to evaluate cardiomyocytes contractile properties: maximum change of sarcomere length during contraction [Shortening (LD-LS)]; and the percentage of shortening; the maximum velocity of shortening (ΔL/Δt).

### Intracellular Ca^2+^ Transient Measurement

Intracellular Ca^2+^ was measured using a dual-excitation, single emission photomultiplier system (IonOptix) (20). Cardiomyocytes were treated with Fura 2-AM (2 μM) at 37°C for 20 min and then exposed to light emitted by a 75 W halogen lamp through either a 340- or 380-nm filter while being stimulated to contract at 14 voltages with a frequency of 1 Hz. Fluorescence emissions were detected. The following parameters were recorded: maximum changes of calcium signal during contraction (ΔFura Ratio); the maximum velocity of shortening (ΔR/Δt); and the percentage of shortening.

### Immunoblotting

Immunoblotting was performed as previously described (19, 23). Heart homogenate proteins were resolved by SDS-PAGE and transferred onto polyvinylidene difluoride membranes (Millipore, Bedford, MA). Rabbit antibodies against phosphor-AMPK (Thr^172^), AMPK, phosphor-PDHE1a, PDHE1a, phosphor-SAPK/JNK (Thr^183^/Tyr^185^), SAPK/JNK, phosphor-NF-κB (Ser^536^), and NF-κB from Cell signaling (Danvers, MA) were purchased and used according to protocols provided by the manufacturer.

### Mitochondrial Respiration Measurements

The Seahorse XF24 was used to measure the oxygen consumption rate (OCR) of isolated cardiomyocytes. Isolated cardiomyocytes and sectioned fresh brain tissues were differentiated in customized Seahorse 24-well. We applied DMEM Medium (Seahorse Bioscience), supplemented with 1 mM pyruvate, 2 mM glutamine, and 10 mM D-glucose. OCR was measured using the Seahorse Bioscience XF24 Extracellular Flux Analyzer (Seahorse Bioscience). Measurements were taken as the cells were incubated sequentially under four conditions: 1) basal levels were measured with no additives; 2) oligomycin (1.5 μM) was added to reversibly inhibit ATP synthase and OXPHOS, showing glycolysis alone; 3) FCCP (1 μM), a mitochondrial uncoupler, was added to induce maximal respiration; and 4) Antimycin A (10 μM), a Complex III inhibitor, was added to obtain non-mitochondrial oxygen consumption background. The Seahorse software was used to plot the results. OCR was normalized to cell number per well.

### ROS Measurements

MitoSOX™ Red (Invitrogen) was used to measure mitochondrial reactive oxygen species (ROS) production. Freshly frozen sections of the left ventricle (LV) were washed by PBS and then incubated within PBS containing 1 μM MitoSOX™ Red mitochondrial superoxide indicator (Invitrogen) for 15 min at 37°C. Slides were rinsed with 1XPBS 3 times every 5 min and counterstained with DiD, a lipophilic fluorescent stain for cardiomyocytes membranes. Images were detected by fluorescence microscopes (excitation at 510, emission at 647 nm). In addition, fresh brain sections were subjected to MitoSOX™ Red staining with the same procedure.

### Amyloid Plaques Deposit Staining

Heparin IV (Fresenius Kabi) for anticoagulation was given by intraperitoneal injection with 1,000 units/kg 10 min before the experiment (22). Six-months old 5XFAD and WT mice underwent anesthesia with 2–3% isoflurane and 100% O_2_. The mice were transcardially perfused with ice-cold 1XPBS. Brains and hearts were rapidly removed, and fixed with 4% paraformaldehyde overnight at 4°C. Subsequently, the hemisphere was subjected to dehydration with 10, 20, and 30% sucrose and then embedded in a cutting temperature compound (Tissue-Tek). Fixed brains and heart tissue was sectioned at 25 and 5 μm thickness setting on a cryostat and postfixes, respectively. After washing with 1XPBS, the sections were blocked win 5% normal donkey serum (Vector Laboratories)/ 0.1% Triton-X/1XPBS for 1 h and incubated with mouse anti-human 6E10 amyloid plaque antibody (Biolegend) diluted in blocking solution overnight at 4°C. After three 1XPBS washes, sections were incubated with secondary antibodies in diluted blocking solution for 1 h at room temperature. Finally, sections were washed with 1XPBS three times and mounted onto slides with DAPI counterstain mounting medium, and observed on an SP8 confocal microscope (Leica). Fiji ImageJ was used to quantify the amyloid plaques load in the hippocampus and cortex.

### Radial Arm Water Maze Behavior Test

Mice are put into the behavioral room in darkness one h prior to the start of the experiment. For 15 consecutive days, the experiment was started around the same time, and each mouse was in the same order. This experiment was carried out in the dark. WT or 5XFAD mice were put into the water maze one at a time at the starting arm. Each day, the platform was placed at the end of the goal arm. For each trial, mice were placed at the center of the starting arm, facing forward. As it started swimming, the experimenter timed for 60 seconds and stopped timing once it reached and climbed up the platform. Errors were counted as: 1) each time the mouse entered the wrong arm, the experimenter gently grabbed its tail and pulled it back to its starting position; 2) if the mouse stayed in the center and did not enter any arm for 15 seconds; 3) if the mouse entered the goal arm without climbing up the platform for 15 seconds; 4) if the mouse did not reach the platform within 60 seconds, while only entering 1 or 2 arms continuously. After the mouse reached the platform, it was allowed to stay on the platform for 30 seconds to gain familiarity with the surrounding. For each trial, the time the mouse took to reach the platform and the number of errors it made were recorded. After the 4th trial, the mouse waited for 30 min to run the 5th trial. After each trial, the water was stirred to avoid the remaining scent that would affect the next mouse. Male and female mice were tested separately and were given one h in between to let the scent dissipate.

### Myocardial Histology

Left ventricular tissue from 5XFAD and WT mice was rapidly excised, cross-sectioned, and fixed in 4% buffered paraformaldehyde. Fixed tissue was then paraffin embedded and sectioned and stained with hematoxylin and eosin (H&E) and Trichrome (24). Slides were then assessed in a blinded fashion with Keyence BZ-X710 All-in-One Fluorescence Microscope under 40X objective magnification power. Fibrosis was analyzed with FIJI Image J as areas stained with blue.

### Statistics Analysis

The analysis results of cardiac function, superoxide accumulation, mitochondrial function, cardiomyocyte functional properties, histological and behavior test, as well as immunoblotting, were expressed as means ± standard error of the means (SEM). Two-tailed Student's *t-*test and one-way ANOVA with Tukey's test were used to perform the comparison of the statistics among a set of samples with Prism 9.0 (GraphPad Software). *P* < 0.05 was considered a significant difference.

## Results

### AD-Model Mice Showed Decreased LV Fractional Shortening and Ejection Fraction With ECG Changes

Fractional shortening (FS) refers to the change between the length of the left ventricle (LV) at the end of systole compared to the end of diastole (25), while ejection fraction (EF) is defined by the amount of blood exiting LV from the beginning until the end of systole (26). Both FS and EF are expressed as percentages and are critical markers of cardiac contractility. In energy-related cardiac dysfunction, both FS and EF significantly decrease (27, 28). Therefore, they are used in our study as part of the indicators of cardiac dysfunction.

**Figure 1:**
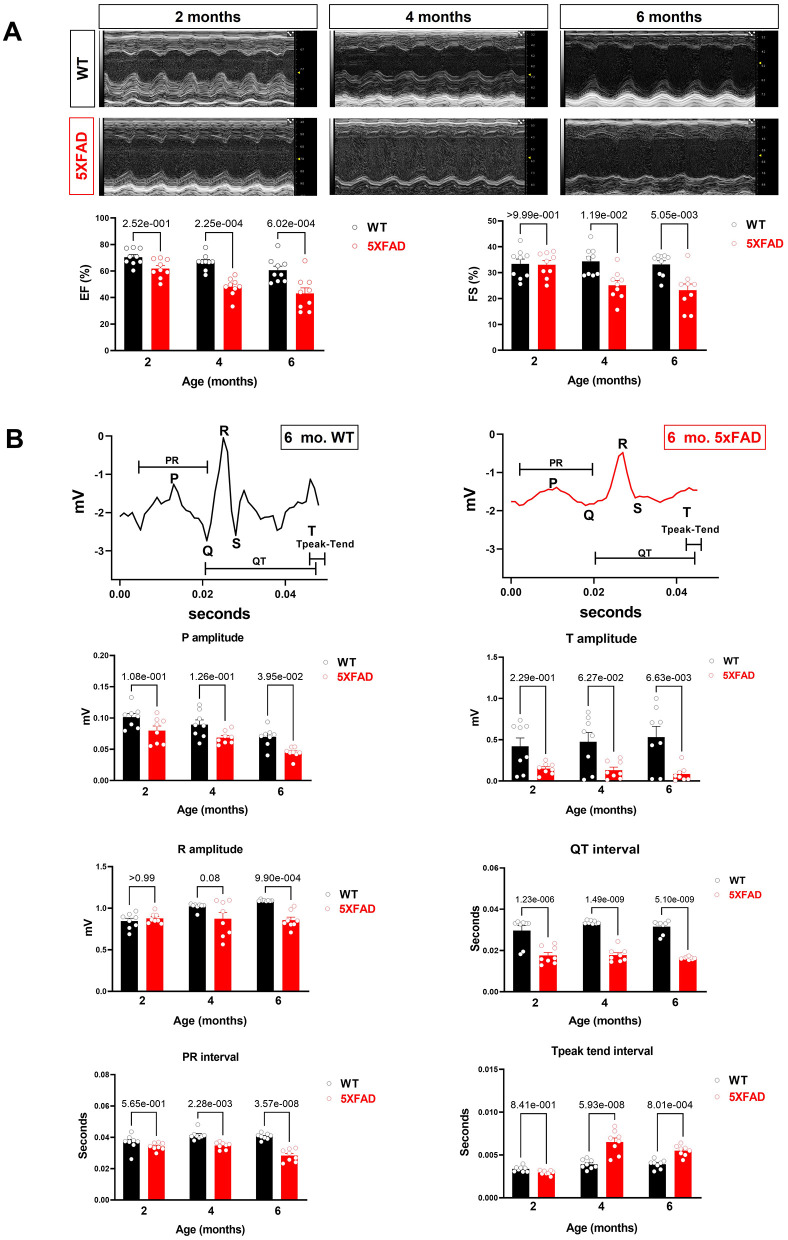
Cardiac systolic dysfunction in 5XFAD compared to WT mice chronologically. **(A)** Echocardiography showed that 5XFAD mice developed progressive cardiac systolic dysfunction over time with reduced left ventricular function as shown by ejection fraction (EF) and fractional shortening (FS). Upper: Representative images of M-mode echocardiography. Lower: Quantification of echocardiography measurements for EF and FS. Biological replicates *N* = 9 for each group. *P*-value was determined by two-way ANOVA with Tukey's post hoc test. **(B)** Electrocardiography (ECG) showed that 5XFAD mice developed a decreased electrical signal over time with a reduced P wave, QRT complex, and the T wave. Upper: Representative images of ECG parameters. Lower: Quantification of ECG measurements. Biological replicates *N* = 8 for each group. *P*-value was determined by two-way ANOVA with Tukey's *post hoc* test.

To assess the changes in cardiac function related to AD, we utilized a strain of mice as a model for AD, 5XFAD, which carried five mutations resulting in early-onset AD around 6-month of age. Both 5XFAD mice and wild-type (WT) counterparts were subjected to echocardiogram for heart function assessment at 2-, 4-, and 6-month of age. Compared to WT, 5XFAD mice showed a reduction in FS which was first observed at 4-month, and progressively further decreased at 6-month (Figure 1A). Similarly, we also observed a progressive reduction in EF by 4-month and 6-month (Figure 1A). These findings demonstrate that the LV of 5XFAD mice had decreased systolic contractile function and suggest a decreased force of contraction.

Along with the decreased contractility, we also observed a reduction in the amplitude of P, R, and T waves on the electrocardiogram in 5XFAD mice compared to WT (Figure 1B). This phenomenon could either be attributed to the heart of 5XFAD mice having defective electrical function a reduced number of cardiomyocytes, which resulted in the dampened electrical signal. Alternatively, past studies have shown amyloidosis of the heart in the extracellular space can make signals more difficult to detect by ECG.

### Cardiomyocytes of AD-Model Mice Exhibited Impaired Extent and Rate of Contraction Associated With Decreased Calcium Influx

**Figure 2:**
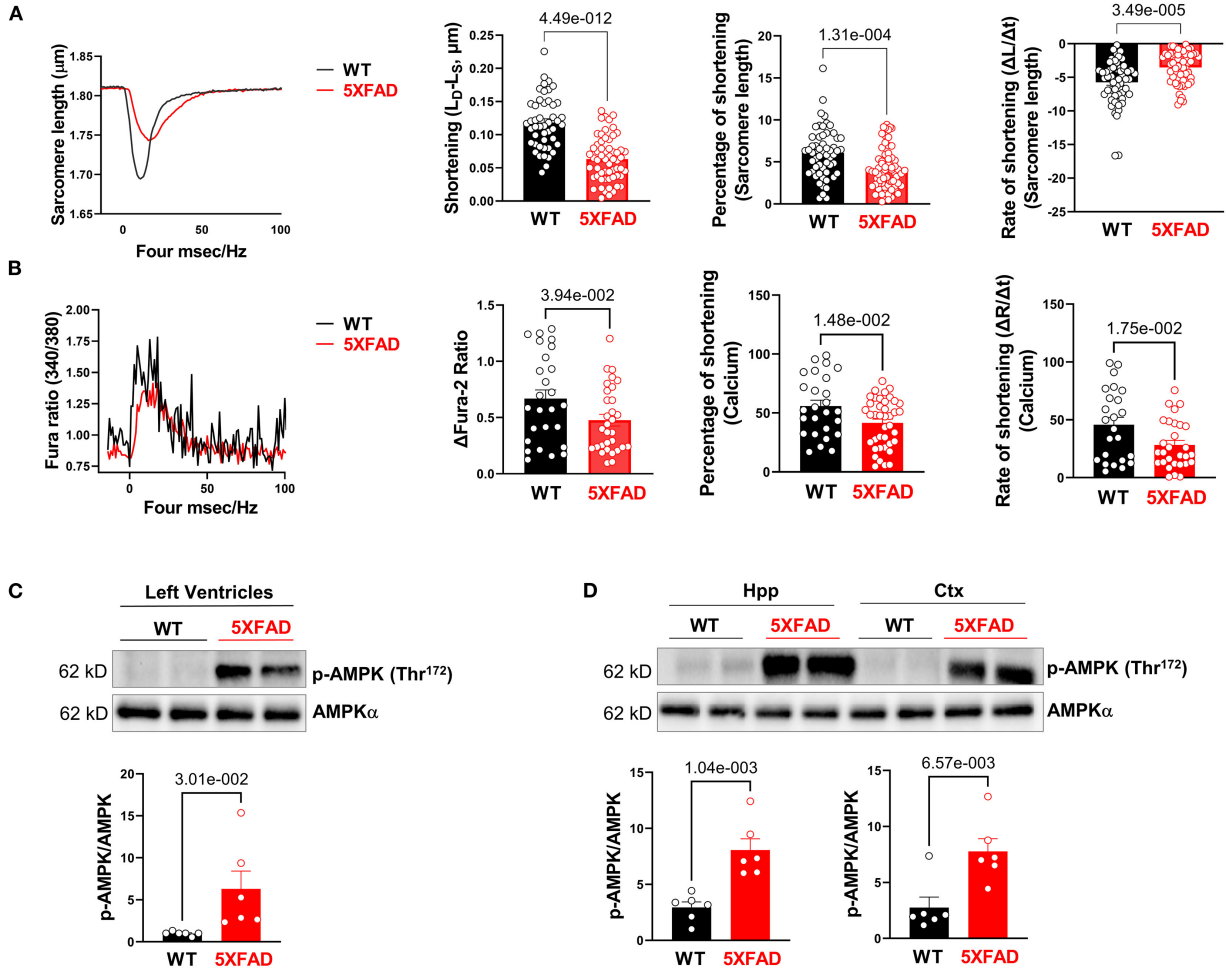
Cardiomyocytes of 5XFAD mice exhibited impaired extend and rate of contraction associated with decreased calcium influx, **(A)** The contractile properties of isolated cardiomyocytes from 5XFAD (6 months) and WT (6 months) hearts. Biological replicates *N* = 47–68 for each group, these cardiomyocytes were isolated from a total of six mice. *P*-value determined by two-tailed students *t*-test. **(B)** The transient calcium signal response of the isolated cardiomyocytes from 5XFAD (6 months) and WT (6 months) hearts. Biological replicates *N* = 24–40 for each group, these cardiomyocytes were isolated from a total of six mice. *P*-value determined by two-tailed students *t*-test. **(C)** Immunoblotting showed the phosphorylation of AMPK at Threonine 172 in left ventricles from 5XFAD (6 months) and WT (6 months) hearts. Biological replicates *N* = 6 for each group. *P*-value determined by two-tailed students *t*-test. **(D)** Immunoblotting showed the phosphorylation of AMPK at Threonine 172 in the hippocampus and cortex from 5XFAD (6 months) and WT (6 months). Biological replicates *N* = 6 for each group. *P*-value determined by two-tailed students *t*-test.

To further assess cardiac function in 5XFAD mice, we isolated cardiomyocytes from both 5XFAD and WT. Cardiomyocyte shortening of sarcomeres during contraction was measured. Viable cardiomyocytes of 5XFAD mice had a significant reduction in sarcomere shortening, represented by both the absolute length of shortening and the percentage of shortening (Figure 2A), which indicated impaired inotropy. The cardiomyocytes of 5XFAD mice also had a slower rate of sarcomere shortening (Figure 2A), indicating impaired contractile function. When examining the course of a duration of contraction as the length of sarcomere changed, the cardiomyocytes of 5XFAD mice had worsened contractility and took longer for them to recover from one contraction event compared to WT (Figure 2A). When observing the transient calcium influx via Fura-2 staining, 5XFAD cardiomyocytes had significantly decreased transient calcium flux noted by calcium shortening peak, the percentage of shortening, and the rate of shortening (Figure 2B). Defective calcium influx with stimulation is consistent with the contractile functional decline in the cardiomyocyte.

Through immunoblotting analysis, we observed a molecular level change that was consistent with contractile dysfunction of 5XFAD cardiomyocytes, that there was an increase in phosphorylation of AMPK at Thr^172^ site in LV (Figure 2C) and both the hippocampus and cortex of the brain (Figure 2D), indicating its increased activation at these sites. AMPK is an enzyme that senses cellular energy levels and is activated during energy-deficient states like ischemia to modulate ATP generation (29). Overall, the contractility function studies as well as this molecular finding directed us to an energy generation-defect hypothesis associated with the development of AD-like symptoms that had a significant effect on the cardiac tissue (5, 30).

### Cardiomyocytes of AD-Model Mice Displayed Impaired Mitochondrial Oxidative Phosphorylation and Enzymatic Function

**Figure 3:**
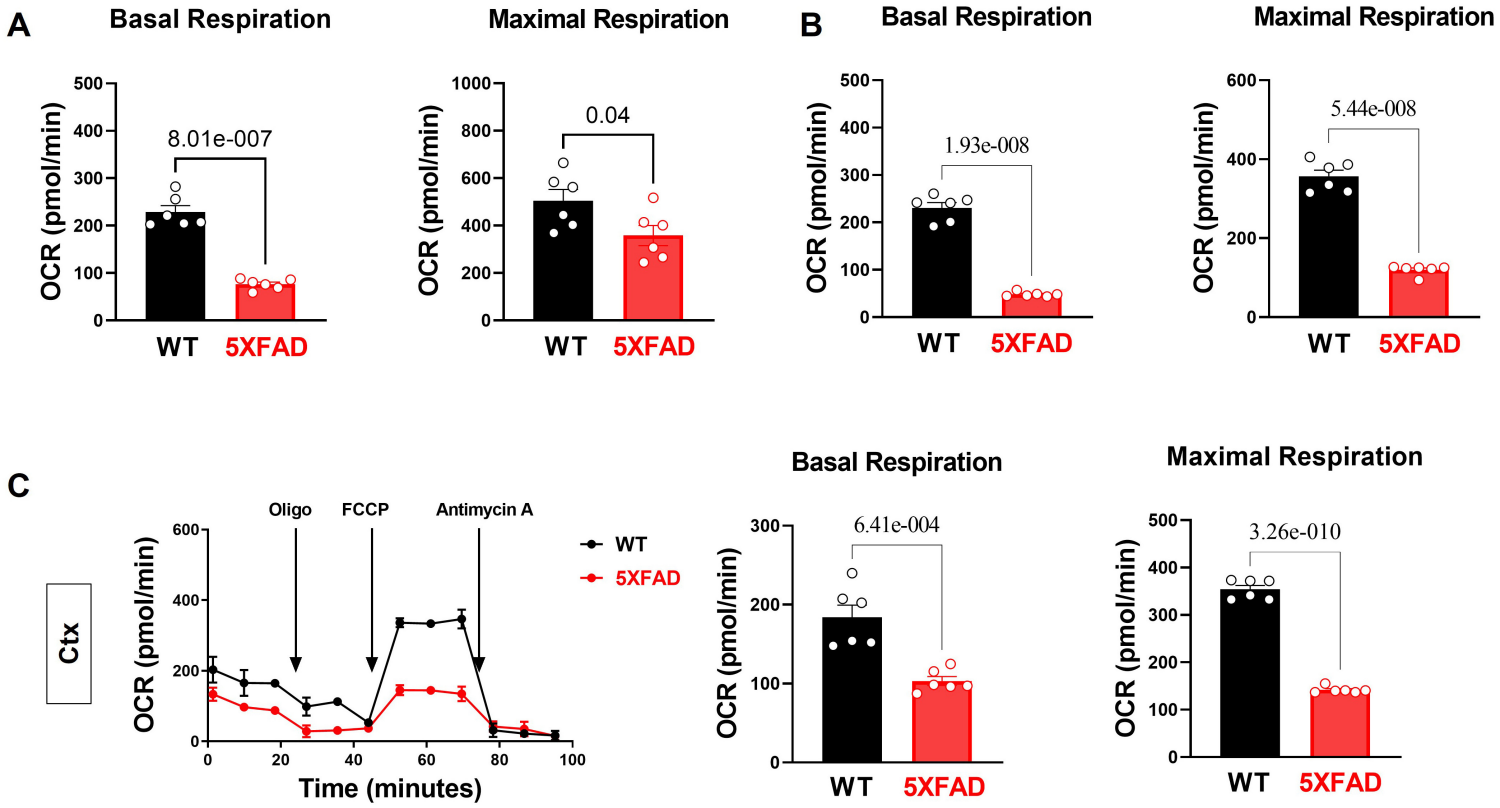
Impairs mitochondrial oxidative phosphorylation in the 5XFAD heart, hippocampus, and cortex. **(A)** Mitochondrial stress assay examined the mitochondrial oxidative phosphorylation (OXPHOS) complexes activity in the heart of 5XFAD (6 months) and WT (6 months) mice demonstrated by measuring the oxygen consumption rate (OCR). *N* = 6 for each group. For each mouse, we collected 9–10 wells using isolated cardiomyocytes for OCR measurement and averaged the results to obtain a single value per mouse. P-value determined by two-tailed students *t*-test. **(B)** Mitochondrial stress assay examined the mitochondrial oxidative phosphorylation (OXPHOS) complexes activity in the hippocampus of 5XFAD (6 months) and WT (6 months) mice demonstrated by measuring the oxygen consumption rate (OCR). *N* = 6 for each group. For each mouse, we collected 1-2 tissue samples for OCR measurement and averaged the results to obtain a single value per mouse. *P*-value determined by twotailed students *t*-test. **(C)** Mitochondrial stress assay examined the mitochondrial oxidative phosphorylation (OXPHOS) complexes activity in the cortex of 5XFAD (6 months) and WT (6 months) mice demonstrated by measuring the oxygen consumption rate (OCR). *N* = 6 for each group. For each mouse, we collected 1-2 tissue samples for OCR measurement and averaged the results to obtain a single value per mouse. *P*-value determined by two-tailed students *t*-test.

Along with the energy deficiency hypothesis, we accessed the function of the mitochondrion in LV tissue in both 5XFAD and WT mice, since most of the energy sources are generated through the process of oxidative phosphorylation in the cardiac tissue (31). Using the Seahorse mito stress test, a baseline oxygen consumption rate (OCR) was obtained. Added inhibitors were used to further assess the mechanism of decreased OCR. Results showed that cardiomyocytes of 5XFAD mice had a lower baseline and maximal OCR compared to WT (Figure 3A). The oxygen consumption is accompanied by ATP generation through mitochondrial oxidative phosphorylation (OXPHOS) activity. Therefore, the results implicated that the cardiomyocytes of 5XFAD mice had decreased ability in ATP production demonstrated by both lower baseline and maximal OCR (Figure 3A). Overall, the cardiomyocytes of 5XFAD with impaired oxygen consumption activity indicate the defective function of mitochondria and the energy production pathway.

Similarly, the brain is also an energy-demanding organ and partially relies on the mitochondria for the energy production (32). In terms of oxygen consumption rate, we observed mitochondrial functional defects in the hippocampus and cortex of 5XFAD mice, with the hippocampus being the most affected region (Figures 3B,C). We also found that the hippocampus had a relatively lower OCR than the cortex at the basal condition, which could be attributed to a lesser number of neurons allocation. The impaired mitochondrial function was associated with decreased basal and maximal OCR, indicating impaired substrate utilization and loss of structural integrity (Figures 3B,C). This result aligned with the recurrent finding that AD-affected brains have a lower capacity for energy generation.

When inspecting the enzymatic function of the mitochondrion in 5XFAD cardiomyocytes and neurons compared to WT, we detected an increase in phosphorylation of PDHE1a at the Ser293 site in LV, hippocampus, and cortex, representing decreased PDH activity (Figures 3D,E). The downregulation of PDH activity could be attributed to phosphorylating inhibition through PDK1 under cellular hypoxia-like conditions, possibly suggesting decreased blood flow or oxygen delivery to cardiomyocytes and brain tissue in 5XFAD mice.

### Cardiac Tissue of AD-Model Mice Displayed Increased Inflammatory Markers

**Figure 4:**
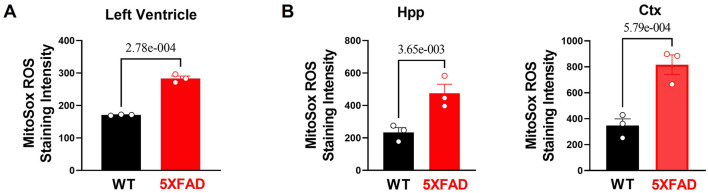
Excessive oxidative stress and provoked cellular proinflammatory signaling in 5XFAD heart and brain. **(A)** MitoSox staining showed increased superoxide accumulation in the heart of 5XFAD (6 months) mice vs. WT (6 months) mice. Biological replicates *N* = 3 for each group. For each mouse, we collected 3–4 tissue sections for superoxide accumulation measurement and averaged the results to obtain a single value per mouse. *P*-value determined by two-tailed students *t*-test. **(B)** MitoSox staining showed increased superoxide accumulation in the hippocampus and cortex of 5XFAD (6 months) mice vs. WT (6 months) mice. Biological replicates *N* = 3 for each group. For each mouse, we collected 3–4 tissue sections for superoxide accumulation measurement and averaged the results to obtain a single value per mouse. *P*-value determined by two-tailed students *t*-test.

Because previous data have demonstrated a mitochondrial defect in cardiomyocytes of 5XFAD mice, we then proceeded to examine the total production of reactive oxygen species (ROS), molecules that upsurge with impaired mitochondrial structure and function and as a potential indicating mechanism for increased cellular inflammation due to oxidative stress (33). MitoSOX™ staining provided a clear visualization of the amount of superoxide accumulation in the mitochondria of both 5XFAD and WT mice. The result showed there was a drastic increase of superoxide production in the tissue of 5XFAD mice in LV, hippocampus, and cortex noted by the increased red foci (Figures 4A,B). This finding showed the functional defective mitochondria of the 5XFAD cardiomyocytes were associated with an increased amount of ROS produced, and hence indicated a greater potential for cellular damage via oxidative stress. A similar result observed in brain tissue confirms that AD progression was associated with an increased inflammatory state in neurons.

When examining other inflammatory cellular protein activities, western-blot data showed the increased activating phosphorylation of NF-kB, a transcription factor that increases the expression of inflammatory mediators. There was also an increase in the activating phosphorylation of JNK, a pro-inflammatory signaling molecule implicated in cellular stress and apoptosis. Overall, the results implicated that increased superoxide with mitochondrial functional deficits in the heart and brain of 5XFAD mice leads to the provoked cellular inflammatory that could cause functional damage in the brain and heart.

### The Tissue of 5XFAD Mice Showed Greater Level of Aβ Deposit

**Figure 5:**
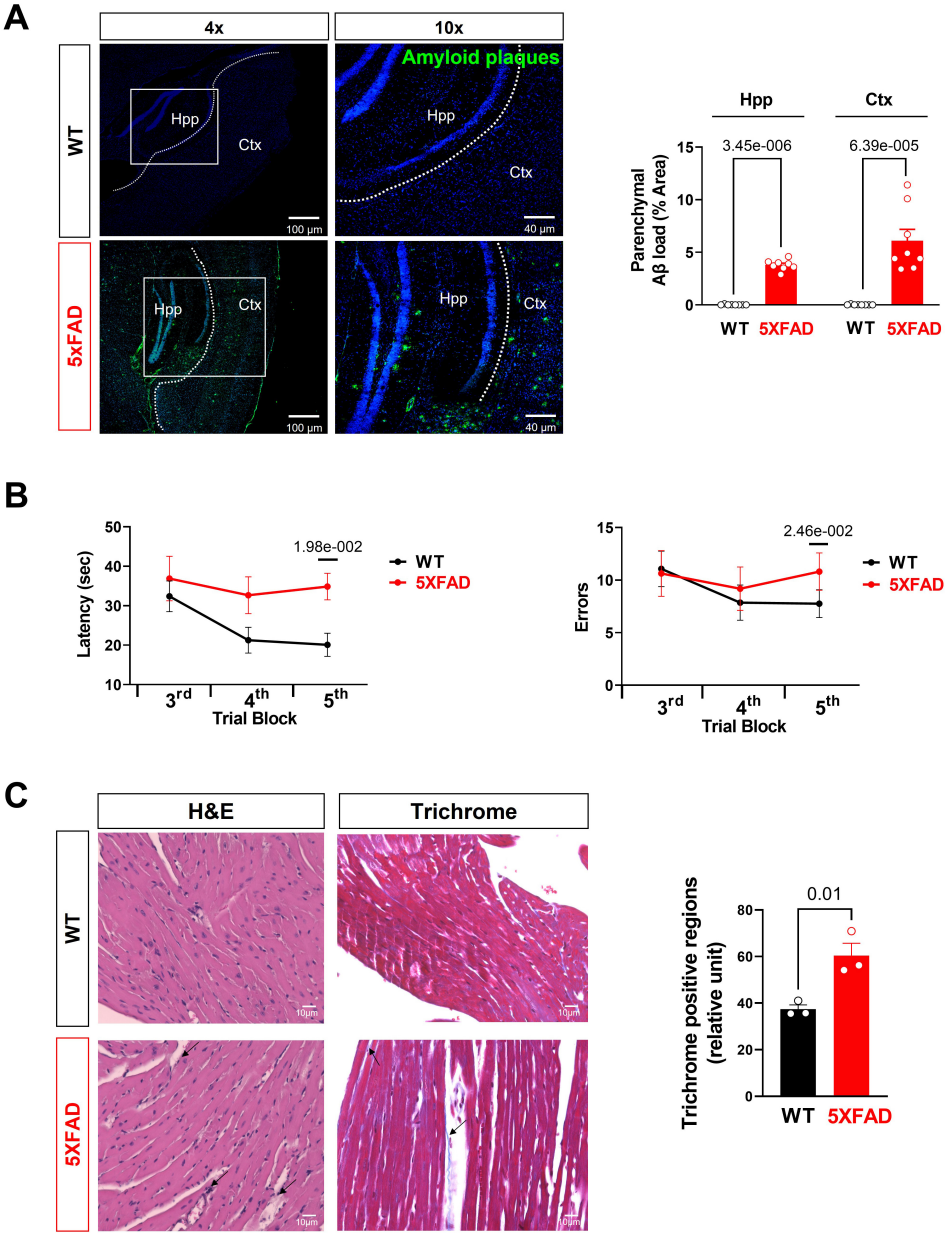
Aβ burden and cognitive function assessment in AD mice. **(A)** Representative images and quantification of Aβ stained with 6E10 antibody in the hippocampus and cortex of 5XFAD (6 months) mice and WT (6 months) mice. Biological replicates *N* = 8 for each group. *P*-value determined by two-tailed students *t*-test. **(B)** Radial arm water maze test showed the latencies to hidden platform and the errors happen in arms in WT (6 months) and 5XFAD (6 months) mice. Biological replicates *N* = 8 for each group. *P*-value determined by two-tailed students *t*-test. **(C)** Myocardial histology analysis showed elevated fibrosis in 5XFAD (6 months) mice heart compared to the WT (6 months) group. Biological replicates *N* = 3 for each group. For each mouse, we collected 4 tissue sections for measurement and averaged the results to obtain a single value per mouse. *P*-value determined by two-tailed students *t*-test. Black arrows highlighted the inflammatory infiltration and fibrosis formation in 5XFAD (6 months) heart H&E staining and Trichrome staining, respectively.

Finally, when directly staining for beta-amyloid deposition, hippocampus and cortex of 5XFAD mice showed significantly increased Aβ deposition compared to WT, consistent with usual findings in AD (Figure 5A). Previous studies have shown an elevated total serum Aβ in AD, predisposing 5XFAD heart tissue to Aβ deposition as one of the most perfused organs (34). Previous studies have shown cardiogenic amyloidosis leads to cardiac diseases such as aortic stenosis and heart failure (35, 36). Due to the systolic dysfunction and mitochondrial defect seen in the cardiomyocytes of 5XFAD mice, we propose that Aβ deposition has direct or indirect effects on the function and viability of cardiomyocytes as it does on neurons shown in the AD.

For confirmation studies that the mice did develop AD-related symptoms, we tested the mice in the Radial Arm Water Maze for their cognitive abilities. The 5XFAD mice showed significantly prolonged time in finding a platform in opaque water after learning compared to the WT group, indicating impaired short-term and spatial memory seen in AD (Figure 5B). They also exhibited significantly more error counts in their attempts to reach the platform, which is another indication of cognitive decline (Figure 5B). Histological examination of LV with H&E staining showed elevated eosinophil count around cardiomyocytes and blood vessels suggesting increased infiltration of inflammatory cells in the LV of the 6 months old 5XFAD mice compared to the WT group (Figure 5C). Moreover, Masson's trichrome staining revealed the increased area of LV fibrosis in the 6 months old 5XFAD heart compared to the WT group, which indicated the cardiac damage followed by fibrosis in AD (Figure 5C).

## Discussion

Research has linked the pathogenesis of the two high energy demanding organs, brain, and heart, through the brain-heart axis (37). Previous studies have shown how the diseased state of one could contribute to the development of abnormalities in the other (38, 39). These findings are consistent with the high comorbidities seen in clinical setting (40). In this study, we strive to establish a clearer connection of how AD pathogenesis is associated with the generation of cardiac dysfunction.

The specific strain of mice used as a model of AD, 5XFAD, was subjected to a series of experiments assessing their cardiac function. First, we demonstrated that 5XFAD mice had lower FS and EF compared to WT. This suggested that AD progression was concurrent with lowered cardiac contractility function, which could lead to decreased overall cardiac output. Next, we found decreased amplitude of P, R, T waves on ECG in 5XFAD mice, suggesting that there were either fewer cells or decreased electrical amplitude per cell. A decrease in energy availability and increased oxidative stress make cells more prone to apoptosis, which eventually is evidenced by the lowered voltages detected by the ECG. On the other hand, cardiac amyloidosis leads to decreased ECG signals.

We isolated cardiomyocytes and assessed their inotropic and chronotropic function. Unsurprisingly, the cardiomyocytes of 5XFAD mice had lowered contractility compared to WT. Our next step was to examine the influx of Ca^2+^, a key ion in cardiomyocyte contraction. Calcium influx triggers the contraction of sarcomeres by triggering Ca^2+^ release from the sarcoplasmic reticulum and subsequently binding to troponin. Our result showed a decrease in Ca^2+^ influx in 5XFAD cardiomyocytes, indicating a decrease in calcium-induced calcium release as a potential mechanism of contractile dysfunction. Overall, the altered Ca^2+^ influx pattern in 5XFAD cardiomyocytes resulted in changes in the sarcomere contraction pattern, with less shortening, and prolonged recovery compared to WT cardiomyocytes.

Subsequently, the molecular finding of increased AMPK activation suggests that the defective contractility of the 5XFAD heart was energy deficiency related. Overall, the decreased contractility function and signs of energy depletion in 5XFAD cardiomyocytes prompted the examination of the biggest energy source generator, the mitochondria. Previous studies demonstrated that mitochondrial dysfunction is implicated in AD and cardiac dysfunction, in which a significant reduction of ATP production, as well as increased oxidative stress, were observed as a result of mitochondrial defect (32, 41). The hypothesis of impaired mitochondrial function was confirmed through the overall decreased basal and maximal OCR. The findings suggest an overall decrease in the efficiency of the mitochondria governing energy resources. Moreover, we observed increased PDH E1a Ser293 phosphorylation presenting in 5XFAD LV, hippocampus, and cortex tissue, indicating the inactivation of the PDH complex. The PDH complex is a mitochondrial multienzyme complex that catalyzes the overall conversion of pyruvate to Acetyl-CoA and CO_2_, linking glycolysis to the TCA cycle (42). Decreased activation of crucial mitochondrial enzymes might indicate the mitochondria is under stress or energy deficit (43). A decrease in the production of Acetyl-CoA, a major energy-generating molecule, due to decreased PDH activity also contributes to an energy-depleted state in heart tissues.

Mitochondrial damage manifests as becoming a “leakier” structure, where normal metabolic and energy-generating substrates cannot effectively pass through the mitochondrial complexes and generate ATP as the product. Moreover, the mitochondrial complexes molecular defects are associated with reactive oxygen species (ROS) production (44). In this process, reactive oxygen species production is elevated, which increases inflammation and causes numerous adverse effects that impact the viability of mitochondria and the cell as a whole (45). As evidenced in the result, we observed increased superoxide content in 5XFAD tissue, indicating increased oxidative stress. We also observed increased activation of inflammatory mediator proteins. Notably, there was increased activating phosphorylation of P65 and JNK. P65 is implicated in the inflammatory process through the NF-kB signaling pathway. JNK phosphorylate c-Jun and is an apoptosis inflammatory mediator, increasing inflammatory gene expression through its downstream effect (46). Overall, the elevated inflammatory markers in 5XFAD heart tissues affect cell function and survival.

Finally, increased Aβ deposition was observed in the cortex and hippocampus of 5XFAD mice, which we propose to be a crucial link between AD and cardiac dysfunction through systematic elevation of Aβ in circulation. There have been studies on how endogenous or exogenous Aβ can lead to numerous cardiovascular disorders such as heart failure and ischemia (47, 48). For example, dysregulation of the BACE1/BACE1-AS/β-amyloid axis was linked to the pathogenesis of heart failure (49); Aβ is contributory in the development of coronary atherosclerosis and is implicated in the pathophysiology of ischemic heart disease and myocardial ischemia/reperfusion injury (30). However, we didn't detect the amyloid plagues in 6-month old 5XFAD mice with immunofluorescence staining (Data not shown) which could be due to the limitation of technique sensitivity and the shortage of time during the pathogenesis process.

We propose that the elevated Aβ in the circulation could affect cardiomyocytes transportation of O_2_ and energy sources such as glucose. It may also interrupt the influx of Ca^2+^ triggered by cardiomyocyte depolarization through interfering with cell membrane structures. Long-term ischemic and energy-shortage states interrupt normal mitochondrial metabolism, leading to a gradual decrease of oxidation phosphorylation rate and defective mitochondrial composition, increasing oxidative stress and inflammation. This cycle of ongoing worsening of cardiomyocytes function due to mitochondrial defect is seen in normal aging, however, genetic mutation created AD-model has a substantially exacerbated aging-related development of cardiac dysfunction.

## Conclusion

In conclusion, AD model 5XFAD mice exhibited impaired mitochondrial function in cardiac tissue compared to WT mice, which showcased the decreased ability to produce energy and increased oxidative stress-related inflammation, resulting in impaired myocardial contractility. The impairment to the mitochondria was demonstrated by decreased baseline and maximal oxidative phosphorylation, diminished critical mitochondrial enzymatic activity of PDH, increased ROS production, and upregulated activation of inflammatory signaling. Overall, these results are proposed to be attributed to the increased Aβ plaque deposition in cardiomyocytes and endothelial cells of 5XFAD caused by the progression of AD symptoms.

## Data Availability Statement

The raw data supporting the conclusions of this article will be made available by the authors, without undue reservation.

## Ethics Statement

The animal study was reviewed and approved by University of South Florida.

## Author Contributions

JM, DR, TL, JF, and YY designed and conducted the study. JM, DR, TL, JF, YY, MK-H, KD, MF, and JL performed data collection and analysis. JM, DR, TL, JF, YY, MK-H, KD, MF, EL, and JL interpreted the data. JM and DR drafted the manuscript. All authors reviewed the results and approved the final version of the manuscript.

## Funding

This work was supported by NIH R21AG071249, R01AG049835, R01GM124108, and R01HL158515.

## Conflict of Interest

The authors declare that the research was conducted in the absence of any commercial or financial relationships that could be construed as a potential conflict of interest.

## Correction note

This article has been corrected with minor changes. These changes do not impact the scientific content of the article.

## Publisher's Note

All claims expressed in this article are solely those of the authors and do not necessarily represent those of their affiliated organizations, or those of the publisher, the editors and the reviewers. Any product that may be evaluated in this article, or claim that may be made by its manufacturer, is not guaranteed or endorsed by the publisher.

## Abbreviations

5XFAD: 5 familial AD mutations
AD: Alzheimer's Disease
AMPK: AMP-activated protein kinase
APP: Amyloid precursor protein
ATP: Adenosine triphosphate
Aβ: beta-amyloid
EF: ejection fraction
FS: fraction shortening
H&E: hematoxylin and eosin
LV: Left ventricle
NF-κB: nuclear factor kappa-light-chain-enhancer of activated B cell
OXPHOS: Oxidative phosphorylation
PDHE1a: Pyruvate Dehydrogenase E1 Subunit Alpha 1
PS1: Presenilin 1
ROS: reactive oxygen species
SAPK/JNK: c-Jun N-terminal protein kinase
WT: wild type.
