# Corrigendum: The cardiac dysfunction caused by metabolic alterations in Alzheimer's disease

**DOI:** 10.3389/fcvm.2025.1578594

**Published:** 2025-08-08

**Authors:** Jiayuan Murphy, Tran Ngoc Van Le, Julia Fedorova, Yi Yang, Meredith Krause-Hauch, Kayla Davitt, Linda Ines Zoungrana, Mohammad Kasim Fatmi, Edward J. Lesnefsky, Ji Li, Di Ren

**Affiliations:** ^1^ Department of Surgery, Morsani College of Medicine, University of South Florida, Tampa, FL, United States; ^2^ Pauley Heart Center, Division of Cardiology, Department of Internal Medicine, Virginia Commonwealth University, Richmond, VA, United States; ^3^ Cardiology Section, Medical Service, Richmond Department of Veterans Affairs Medical Center, Richmond, VA, United States

**Keywords:** Alzheimer's Disease, cardiac dysfunction, mitochondrial deficits, metabolic regulation, glucose metabolic alterations

In the published article, there was an error in the legend for Figure 2A and 2B as published. In Figure 2A the data represents contractility measurements from isolated cardiomyocytes, not from whole heart tissue, the reported number of biological replicates did not accurately reflect the number of individual cardiomyocytes analyzed per group. In Figure 2B the data represents calcium flux measurements from isolated cardiomyocytes, not from whole heart tissue, the reported number of biological replicates did not accurately reflect the number of individual cardiomyocytes analyzed per group. The corrected legend appears below.

**Figure 2. Cardiomyocytes of 5XFAD mice exhibited impaired extend and rate of contraction associated with decreased calcium influx, (A)** The contractile properties of isolated cardiomyocytes from 5XFAD (6 months) and WT (6 months) hearts. Biological replicates *N* = 47–68 for each group, these cardiomyocytes were isolated from a total of six mice. *P*-value determined by two-tailed students *t*-test. **(B)** The transient calcium signal response of the isolated cardiomyocytes from 5XFAD (6 months) and WT (6 months) hearts. Biological replicates *N* = 24–40 for each group, these cardiomyocytes were isolated from a total of six mice. *P*-value determined by two-tailed students *t-*test. **(C)** Immunoblotting showed the phosphorylation of AMPK at Threonine 172 in left ventricles from 5XFAD (6 months) and WT (6 months) hearts. Biological replicates *N* = 6 for each group. *P*-value determined by two-tailed students *t*-test. **(D)** Immunoblotting showed the phosphorylation of AMPK at Threonine 172 in the hippocampus and cortex from 5XFAD (6 months) and WT (6 months). Biological replicates *N* = 6 for each group. *P*-value determined by two-tailed students *t-*test.

In the published article, there was an error in Figure 1B as published. The data in R amplitude represents both technical replicates and biological replicates. Therefore, there are more datapoints than the reported number of biological replicates *N* = 8 per group**.** The PR interval in Figure 1B was inadvertently duplicated in the final manuscript. This error appears to have been introduced during the final proof submission due to a technical issue when transferring files via different system. The corrected Figure 1 and its caption appear below.

**Figure 1:**
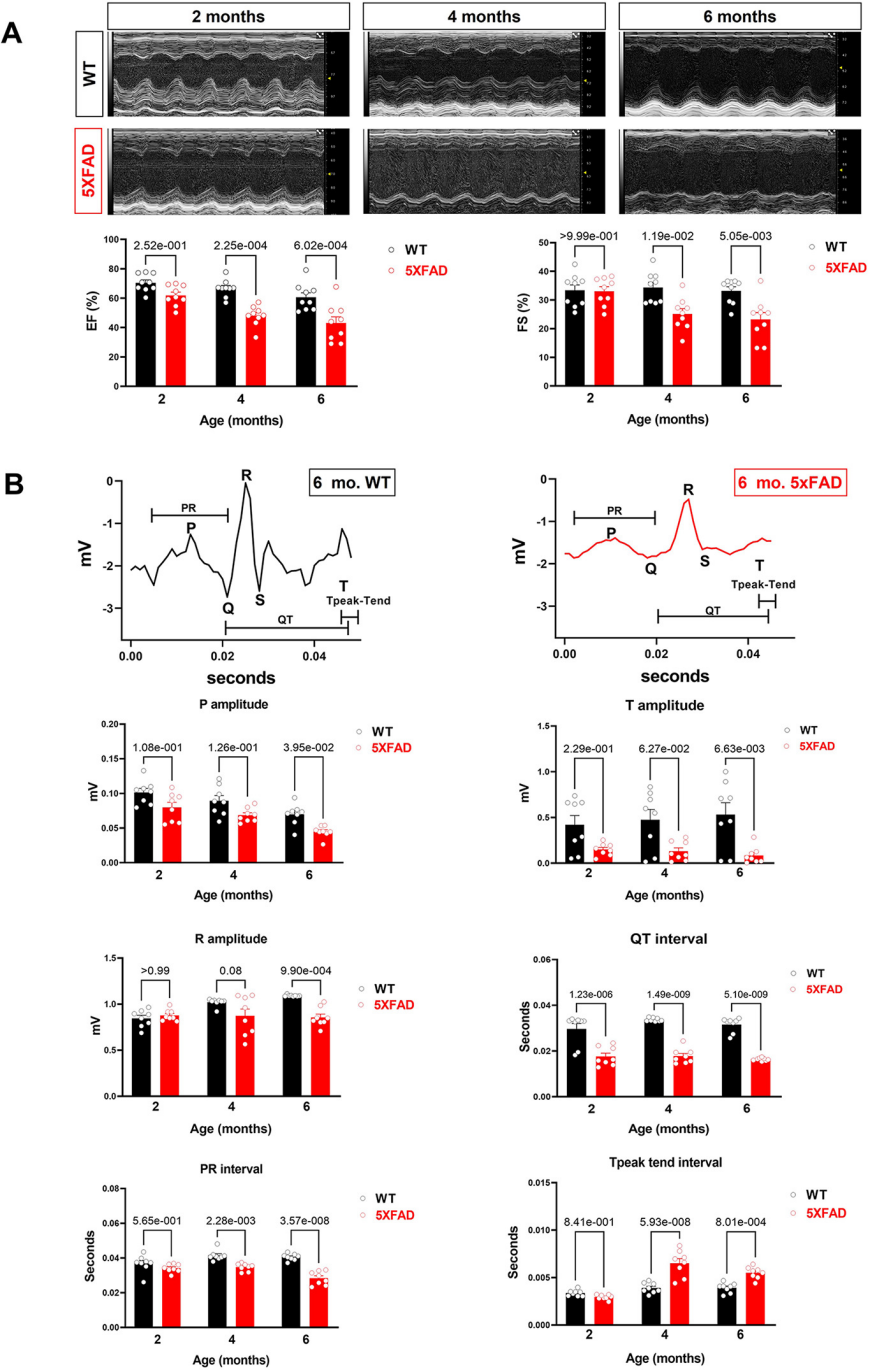
Cardiac systolic dysfunction in 5XFAD compared to WT mice chronologically. **(A)** Echocardiography showed that 5XFAD mice developed progressive cardiac systolic dysfunction over time with reduced left ventricular function as shown by ejection fraction (EF) and fractional shortening (FS). Upper: Representative images of M-mode echocardiography. Lower: Quantification of echocardiography measurements for EF and FS. Biological replicates *N* = 9 for each group. *P*-value was determined by two-way ANOVA with Tukey's *post hoc* test. **(B)** Electrocardiography (ECG) showed that 5XFAD mice developed a decreased electrical signal over time with a reduced P wave, QRT complex, and the T wave. Upper: Representative images of ECG parameters. Lower: Quantification of ECG measurements. Biological replicates *N* = 8 for each group. *P*-value was determined by two-way ANOVA with Tukey's *post hoc* test.

**Figure 3:**
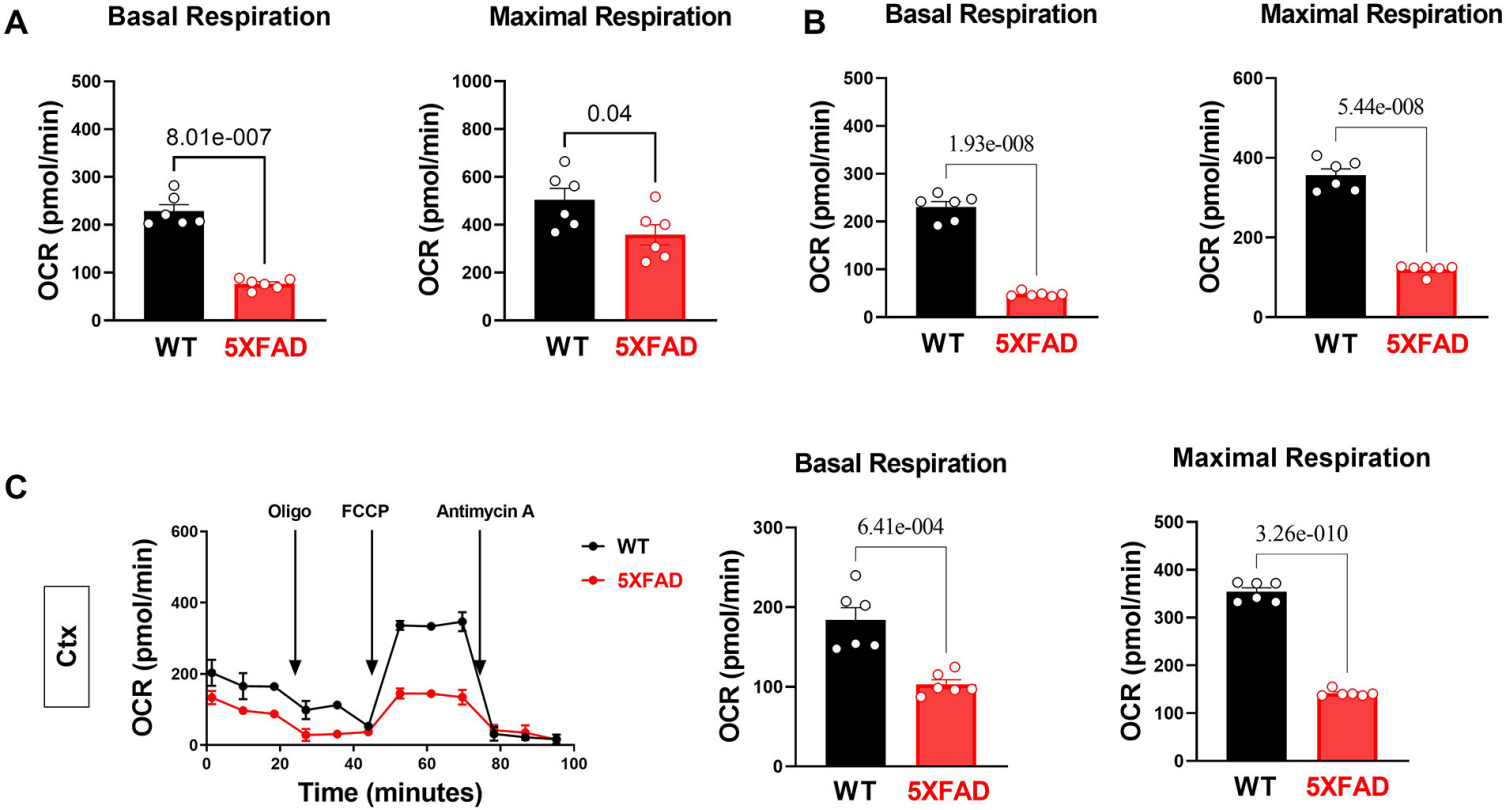
Impairs mitochondrial oxidative phosphorylation in the 5XFAD heart, hippocampus, and cortex. **(A)** Mitochondrial stress assay examined the mitochondrial oxidative phosphorylation (OXPHOS) complexes activity in the heart of 5XFAD (6 months) and WT (6 months) mice demonstrated by measuring the oxygen consumption rate (OCR). *N* = 6 for each group. For each mouse, we collected 9–10 wells using isolated cardiomyocytes for OCR measurement and averaged the results to obtain a single value per mouse. *P*-value determined by two-tailed students *t*-test. **(B)** Mitochondrial stress assay examined the mitochondrial oxidative phosphorylation (OXPHOS) complexes activity in the hippocampus of 5XFAD (6 months) and WT (6 months) mice demonstrated by measuring the oxygen consumption rate (OCR). *N* = 6 for each group. For each mouse, we collected 1–2 tissue samples for OCR measurement and averaged the results to obtain a single value per mouse. *P*-value determined by two-tailed students *t*-test. **(C)** Mitochondrial stress assay examined the mitochondrial oxidative phosphorylation (OXPHOS) complexes activity in the cortex of 5XFAD (6 months) and WT (6 months) mice demonstrated by measuring the oxygen consumption rate (OCR). *N* = 6 for each group. For each mouse, we collected 1–2 tissue samples for OCR measurement and averaged the results to obtain a single value per mouse. *P*-value determined by two-tailed students *t*-test.

**Figure 4:**
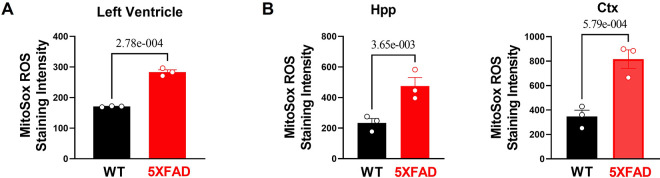
Excessive oxidative stress and provoked cellular proinflammatory signaling in 5XFAD heart and brain. **(A)** MitoSox staining showed increased superoxide accumulation in the heart of 5XFAD (6 months) mice vs. WT (6 months) mice. Biological replicates *N* = 3 for each group. For each mouse, we collected 3–4 tissue sections for superoxide accumulation measurement and averaged the results to obtain a single value per mouse. *P*-value determined by two-tailed students *t*-test. **(B)** MitoSox staining showed increased superoxide accumulation in the hippocampus and cortex of 5XFAD (6 months) mice vs. WT (6 months) mice. Biological replicates *N* = 3 for each group. For each mouse, we collected 3–4 tissue sections for superoxide accumulation measurement and averaged the results to obtain a single value per mouse. *P*-value determined by two-tailed students *t*-test.

**Figure 5:**
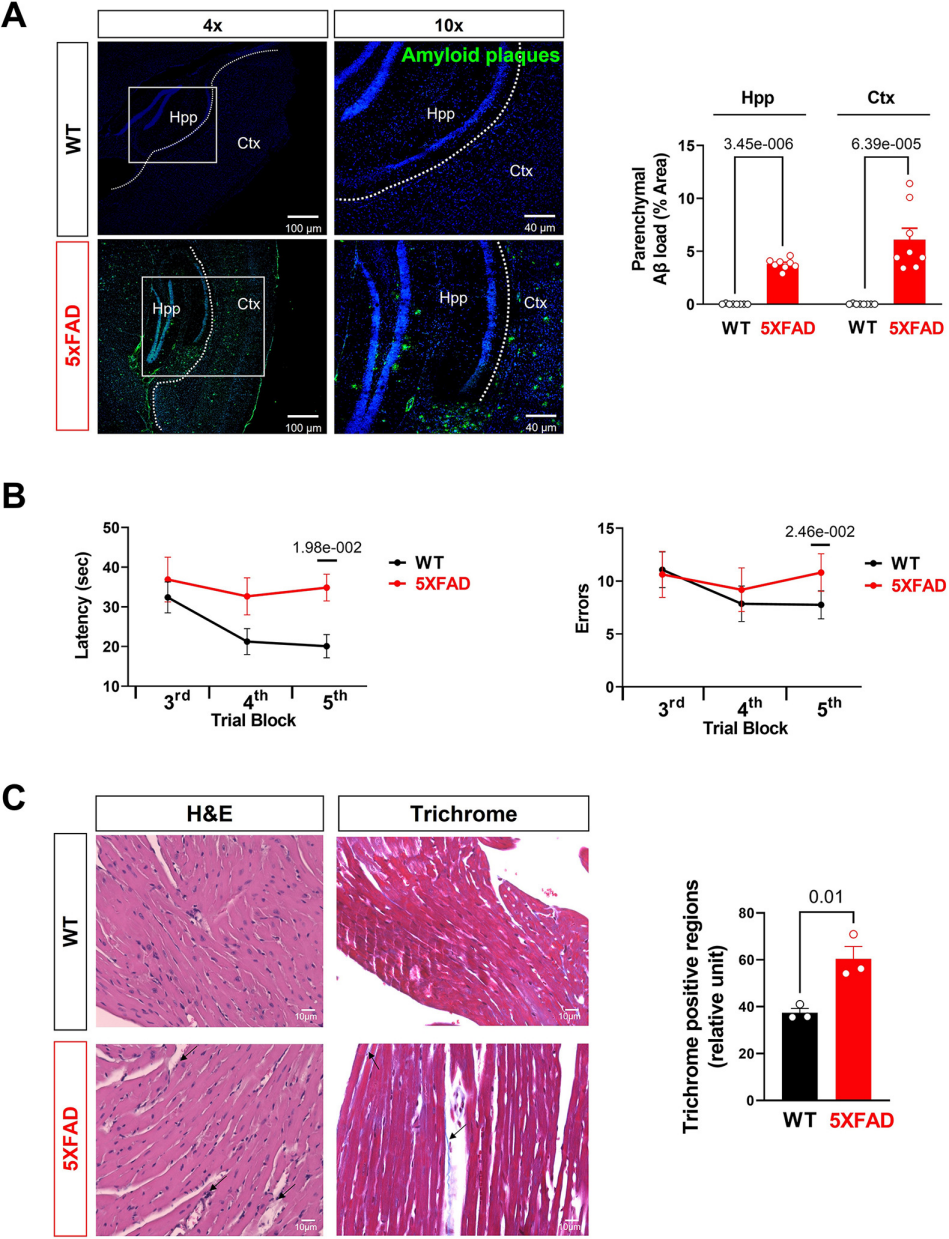
Aβ burden and cognitive function assessment in AD mice. **(A)** Representative images and quantification of A*β* stained with 6E10 antibody in the hippocampus and cortex of 5XFAD (6 months) mice and WT (6 months) mice. Biological replicates *N* = 8 for each group. *P*-value determined by two-tailed students *t*-test. **(B)** Radial arm water maze test showed the latencies to hidden platform and the errors happen in arms in WT (6 months) and 5XFAD (6 months) mice. Biological replicates *N* = 8 for each group. *P*-value determined by two-tailed students *t*-test. **(C)** Myocardial histology analysis showed elevated fibrosis in 5XFAD (6 months) mice heart compared to the WT (6 months) group. Biological replicates *N* = 3 for each group. For each mouse, we collected 4 tissue sections for measurement and averaged the results to obtain a single value per mouse. *P*-value determined by two-tailed students *t-*test. Black arrows highlighted the inflammatory infiltration and fibrosis formation in 5XFAD (6 months) heart H&E staining and Trichrome staining, respectively.

In the published article, there was an error in Figure 3A, 3B and 3C as published. In Figure 3A the data in Basal Respiration and Maximal Respiration represent both biological dependent replicates (Left ventricular tissues) and biological independent replicates (different mice). Therefore, there are more datapoints than the reported number of biological replicates *N* = 6 per group. In Figure 3B the data in Basal Respiration and Maximal Respiration represent both biological dependent replicates (Hippocampus tissues) and biological independent replicates (different mice). Therefore, there are more datapoints than the reported number of biological replicates *N* = 6 per group. In Figure 3C Ctx OCR was inadvertently duplicated. The data in Basal Respiration and Maximal Respiration represent both biological dependent replicates (Cortex tissues) and biological independent replicates (different mice). Therefore, there are more datapoints than the reported number of biological replicates *N* = 6 per group**.** The corrected Figure 3 and its caption appear below.

In the published article, there was an error in Figure 4A and 4B as published. The data in left ventricular, hippocampus, and cortex represent both biological dependent replicates (multiple imaging sections) and biological independent replicates (different mice). Therefore, there are more datapoints than the reported number of biological replicates *N* = 3 per group**.** The corrected Figure 4 and its caption appear below.

In the published article, there was an error in Figure 5C as published. The data in Figure 5C represent both biological dependent replicates (multiple imaging sections) and biological independent replicates (different mice). Therefore, there are more datapoints than the reported number of biological replicates *N* = 3 per group**.** The corrected Figure 5 and its caption appear below.

The authors apologize for these errors and state that this does not change the scientific conclusions of the article in any way. The original article has been updated.

